# Pre-Antiretroviral Therapy Serum Selenium Concentrations Predict WHO Stages 3, 4 or Death but not Virologic Failure Post-Antiretroviral Therapy

**DOI:** 10.3390/nu6115061

**Published:** 2014-11-13

**Authors:** Rupak Shivakoti, Nikhil Gupte, Wei-Teng Yang, Noluthando Mwelase, Cecilia Kanyama, Alice M. Tang, Sandy Pillay, Wadzanai Samaneka, Cynthia Riviere, Sima Berendes, Javier R. Lama, Sandra W. Cardoso, Patcharaphan Sugandhavesa, Richard D. Semba, Parul Christian, Thomas B. Campbell, Amita Gupta, the NWCS 319 and PEARLS study team

**Affiliations:** 1 Department of Medicine, Johns Hopkins University School of Medicine, Baltimore, MD 21287, USA; E-Mails: rshivak1@jhmi.edu (R.S.); ngupte1@jhmi.edu (N.G.); weiteng.yang@gmail.com (W.-T.Y.); 2 Department of Medicine, University of Witwatersrand, Johannesburg 2050, South Africa; E-Mail: tmwelase@witshealth.co.za; 3 University of North Carolina Lilongwe, Lilongwe, Private Bag A-104, Malawi; E-Mail: ckanyama@unclilongwe.org; 4 Department of Public Health and Community Medicine, Tufts University School of Medicine, Boston, MA 02111, USA; E-Mail: Alice.Tang@tufts.edu; 5 Durban International Clinical Research Site, Durban University of Technology, Durban 4001, South Africa; E-Mail: Pillay@ukzn.ac.za; 6 University of Zimbabwe Clinical Research Centre, Harare 999, Zimbabwe; E-Mail: wsamaneka@uzcrc.co.zw; 7 Les Centres GHESKIO, Port-Au-Prince, HT-6110, Haiti; E-Mail: criviere@gheskio.org; 8 International Public Health Department, Liverpool School of Tropical Medicine, Liverpool L3 5QA, UK; E-Mail: sima.berendes@googlemail.com; 9 Asociacion Civil Impacta Salud y Educacion, Lima, 4, Peru; E-Mail: jrlama@impactaperu.org; 10 STD/AIDS Clinical Research Laboratory, Instituto de Pesquisa Clinica Evandro Chagas, Fundacao Oswaldo Cruz, Rio de Janeiro 21045-900, Brazil; E-Mail: sandra.wagner@ipec.fiocruz.br; 11 Research Institute for Health Sciences, Chiang Mai 50200, Thailand; E-Mail: patcharaphan@rihes-cmu.org; 12 Department of Ophthalmology, Johns Hopkins University School of Medicine, Baltimore, MD 21287, USA; E-Mail: rdsemba@jhmi.edu; 13 Department of International Health, Johns Hopkins Bloomberg School of Public Health, Baltimore, MD 21205, USA; E-Mail: pchrist1@jhu.edu; 14 Department of Medicine, Division of Infectious Diseases, University of Colorado School of Medicine, Aurora, CO 80045, USA; E-Mail: Thomas.Campbell@ucdenver.edu

**Keywords:** HIV, selenium, antiretroviral therapy, nutrition, treatment failure, cohort studies

## Abstract

A case-cohort study, within a multi-country trial of antiretroviral therapy (ART) efficacy (Prospective Evaluation of Antiretrovirals in Resource Limited Settings (PEARLS)), was conducted to determine if pre-ART serum selenium deficiency is independently associated with human immunodeficiency virus (HIV) disease progression after ART initiation. Cases were HIV-1 infected adults with either clinical failure (incident World Health Organization (WHO) stage 3, 4 or death by 96 weeks) or virologic failure by 24 months. Risk factors for serum selenium deficiency (<85 μg/L) pre-ART and its association with outcomes were examined. Median serum selenium concentration was 82.04 μg/L (Interquartile range (IQR): 57.28–99.89) and serum selenium deficiency was 53%, varying widely by country from 0% to 100%. In multivariable models, risk factors for serum selenium deficiency were country, previous tuberculosis, anemia, and elevated C-reactive protein. Serum selenium deficiency was not associated with either clinical failure or virologic failure in multivariable models. However, relative to people in the third quartile (74.86–95.10 μg/L) of serum selenium, we observed increased hazards (adjusted hazards ratio (HR): 3.50; 95% confidence intervals (CI): 1.30–9.42) of clinical failure but not virologic failure for people in the highest quartile. If future studies confirm this relationship of high serum selenium with increased clinical failure, a cautious approach to selenium supplementation might be needed, especially in HIV-infected populations with sufficient or unknown levels of selenium.

## 1. Introduction

There are around 35 million people living with Human immunodeficiency virus (HIV) infection in both developing and developed countries [1]. Access to antiretroviral therapy (ART), which can control but not cure the infection, has increased 30-fold since 2003 [1]. As treatment needs to continue for life and the number of people on ART is expected to rise further, with 7.1 million still lacking access to ART [1], it is important to understand ways to maximize treatment efficacy. Clinical treatment failure (World Health Organization (WHO) defined stage 3, 4 or death) and virologic treatment failure have been attributed to a variety of causes including malnutrition and micronutrient deficiencies [2,3,4]. Serum selenium deficiency has been associated with higher morbidity and mortality among ART-naïve HIV-infected people in multiple studies but there is mixed data to date on whether selenium supplementation has benefits in HIV population [5,6,7,8,9,10]. Whether selenium deficiency is associated with adverse treatment outcomes in the era of ART remains unanswered. In addition, selenium has a U-shaped link with other health outcomes including cancer and all-cause mortality [11], but whether or not that is relevant to HIV outcomes post-ART initiation is not known.

Selenium is an essential micronutrient and an essential component of the selenocysteine amino acid that is incorporated into 25 different selenoproteins [12]. Most of the well-studied selenoproteins, such as glutathione peroxidase (Gpx) and thioredoxin reductase (Txnrd), are antioxidants while other selenoproteins regulate metabolism or tissue development [13]. Antioxidants, such as selenoproteins, can counteract the oxidative stress and subsequent damage to DNA, proteins and lipids caused by pro-oxidants. This is important in the context of HIV infection as oxidative stress is increased during both HIV infection and ART treatment [14]. In addition, the selenoproteins Gpx and Txnrd1 have been shown to be important during HIV infection by decreasing viral activation and negatively regulating the HIV Tat protein, respectively [15,16].

Selenium concentration varies by geography [17] and has a bi-directional relationship with inflammation and the acute phase response [11,18]. Low concentrations of selenium have been linked with disorder of various tissues and diseases [11]. However, higher concentrations of selenium can also be detrimental as seen in US populations where a non-linear U-shaped relationship with cancer and all-cause mortality has been observed [11]. Studies to date in HIV-infected adults have not assessed the association between serum selenium deficiency pre-ART and clinical outcomes post-ART, while many pre-ART era studies did not adjust for inflammation and other confounders [11,19].

The objective of our study was to assess the risk factors for serum selenium deficiency pre-ART (baseline) and to determine if baseline serum selenium deficiency was independently associated with treatment outcomes after controlling for potential confounders including inflammation. To address these questions, we conducted a case-cohort study of ART naïve HIV-infected patients who initiated ART in the “Prospective Evaluation of Antiretrovirals in Resource Limited Settings (PEARLS)” trial [20]. Based on existing literature of HIV-infected ART-naïve individuals, we hypothesized that pre-ART serum selenium deficiency would be associated with greater treatment failure. Interestingly, we find that high levels of serum selenium concentrations, more so than serum selenium deficiency, were associated with adverse treatment outcomes and our results could have implications for selenium supplementation studies.

## 2. Methods

### 2.1. Study Population

We performed a nested case-cohort study within the PEARLS trial [20], a randomized clinical trial of the AIDS Clinical Trial Group (ACTG A5175, clinicaltrials.gov NCT00084136). 1571 HIV-1 infected adults (≥18 years old) with CD4+ T cell count less than 300 cells/mm^3^ were enrolled and followed between May 2005 and May 2010. Participants were randomly allocated with equal probability to one of three different ART regimens: (A) efavirenz plus twice-daily lamivudine-zidovudine; (B) atazanavir plus didanosine EC and emtricitabine all given once daily; (C) and efarivenz plus emtricitabine-tenoforvir-DF once daily. Participants were from nine different countries: Brazil (*n* = 231), Haiti (*n* = 100), India (*n* = 255), Malawi (*n* = 221), Peru (*n* = 134), South Africa (*n* = 210), Thailand (*n* = 100), United States (*n* = 210) and Zimbabwe (*n* = 110). Participants who were pregnant or had an acute illness or other comorbidities, as described in detail elsewhere [20], were excluded from the study.

**Figure 1:**
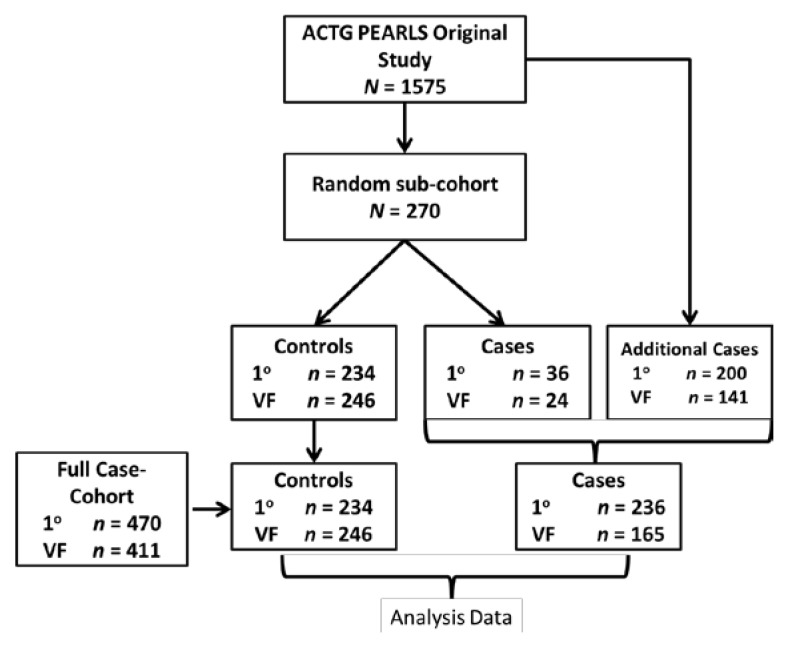
Case-cohort design for primary clinical treatment failure and secondary virologic failure. The case-cohort analysis consisted of the random sub-cohort (random subsample from full cohort) and any additional cases from the full cohort that met the outcome definition. Our primary outcome (1°) was defined as incident WHO stage 3 or 4 event or death (*n* = 234 controls and *n* = 236 cases, total *N* = 470) within 96 weeks post-ART initiation. Secondary outcomes was virologic failure (VF) (*n* = 246 controls and *n* = 165 cases, total *N* = 411), defined as 2 successive plasma HIV-1 RNA levels >1000 copies/mL at or after 16 weeks post-ART initiation. The subcohort was used to determine prevalence and characteristics associated with serum selenium deficiency. The full-case cohort (analysis data) was used to study the association of baseline serum selenium concentrations with treatment outcome.

We selected the case-cohort design for this study as this allows for prevalence estimation and assessment of multiple outcomes [21,22]. The case-cohort was composed of a random subcohort of 270 participants along with the remaining cases in PEARLS that were not sampled as part of the subcohort (Figure 1). Cases were defined as either primary treatment failure or secondary treatment failure. Primary case outcome for this study was clinical treatment failure (incident WHO stage 3, 4 or death), and secondary case outcome was virologic failure (HIV-1 RNA levels ≥1000 copies/mL for two successive visits at ≥16 weeks after study entry). The random subcohort was selected by stratified random sampling with 30 participants from each of the nine countries. The total number of people in the case-cohort was dependent on whether the outcome of interest was primary (treatment failure, *n* = 470), or secondary (virologic failure, *n* = 411) (Figure 1).

### 2.2. Ethics Statement

Institutional review board and ethics committees at each participating institution and the Johns Hopkins University School of Medicine granted approval for this study. Appropriate guidelines from the United States Department of Health and Human Services for human experimentation were followed and participants granted written informed consent for the study.

### 2.3. Data Collection and Laboratory Analysis

Clinical history was measured at entry (pre-ART) and at weeks 2, 4, 8 and every four weeks after that through week 24, and every eight weeks through week 96. Diagnostic criteria were standardized across the different clinical sites. Body mass index (BMI) was measured at baseline and serum was collected at baseline and stored at −80 °C. Plasma HIV-1 viral load was assessed using Roche Amplicor Monitor Assay (v1.5, Roche Molecular Diagnostics, Branchburg, NJ, USA). Baseline serum selenium concentration was measured using a Perkin-Elmer Analyst 600 graphite furnace atomic absorption spectrometer at Johns Hopkins University and were run in batches. Serum selenium deficiency was defined as serum concentrations of <85 μg /L based on previous studies of HIV and selenium [23,24,25]. C-reactive protein (CRP) was measured using an enzyme-linked immunosorbent assay (ELISA) kit (R & D Systems). Serum albumin, hemoglobin and CD4 cell counts were also assessed at site laboratories that were all externally quality assured as part of the National Institute of Health (NIH) Division of AIDS and ACTG Network lab quality assurance [20].

### 2.4. Statistical Analysis

The random subcohort was used for prevalence estimates of baseline serum selenium deficiency by various baseline characteristics. Differences in serum selenium deficiency prevalence by covariates were tested using the Wilcoxon’s rank sum test for continuous variables and Fisher’s exact test for categorical variables. CRP and viral load variables were log-transformed. Odds ratios (OR) and 95% confidence intervals (CI) of serum selenium deficiency at baseline were estimated by covariates using univariable and multivariable logistic regression. Age, gender and albumin were forced in multivariable models due to evidence of association with selenium in past studies [19,26] and also because they had a trend of association (*p* < 0.1) in univariable models. BMI was forced in the model to control for malnutrition.

Associations between outcomes and serum selenium concentrations adjusting for other covariates were estimated for the full case-cohort using univariable and multivariable Cox proportional hazard models stratified by treatment and country for clinical failure and by country for virologic failure. Stratifying by treatment in addition to country for virologic failure models did not change the results (data not shown). Barlow weighting [21] and robust standard errors were used in the Cox models to account for the stratified design of the study.

Selenium was categorized as either normal or selenium deficient based on serum cutoffs (<85 μg/L) or as quartiles of serum selenium for univariable and multivariable analyses of clinical treatment failure and virologic failure. Multivariable model 1 was used to determine association of selenium status (binary or quartile) with clinical failure after adjusting for gender, age, BMI, CD4 count, viral load, hemoglobin and albumin while multivariable model 2 also adjusted for inflammation (CRP). Multivariable model 3 was used to determined association of selenium status with virologic failure after adjusting for gender, age, BMI, CD4 count, viral load, treatment arm while multivariable model 4 also adjusted for CRP. Further adjusting for albumin and hemoglobin in model 3 and 4 did not affect the results and a simpler model is presented given that previous studies do not usually include these variables in virologic failure models. Race was collinear with country and was not included in multivariable models. STATA version 13 (Stata Corp, College Station, TX, USA) and S-plus Tibco Spotfire version 7.2 (TIBCO, Palo Alto, CA, USA) were used for data analysis. Forest plots were created using GraphPad Prism Software version 5 (GraphPad software Inc., La Jolla, CA, USA).

## 3. Results

### 3.1. Description of Study Population

As shown in Figure 1, the primary (clinical treatment failure as outcome) case-cohort comprised of 470 individuals with 234 controls and 236 cases. The secondary (virologic failure as outcome) case-cohort comprised of 411 individuals with 246 controls and 165 cases. Serum selenium concentrations were available for 88% (*n* = 413) of primary case-cohort and 93% (*n* = 381) of the secondary case-cohort participants. There were no differences in the characteristics (age, gender, BMI) of participants with missing selenium concentrations compared to the participants with available ones. The majority of missing data, 78% of primary case-cohort and 52% of secondary case-cohort, were samples from India, which could not be assayed due to regulatory difficulties with exporting samples.

### 3.2. Risk Factors for Baseline Serum Selenium Deficiency

**Figure 2:**
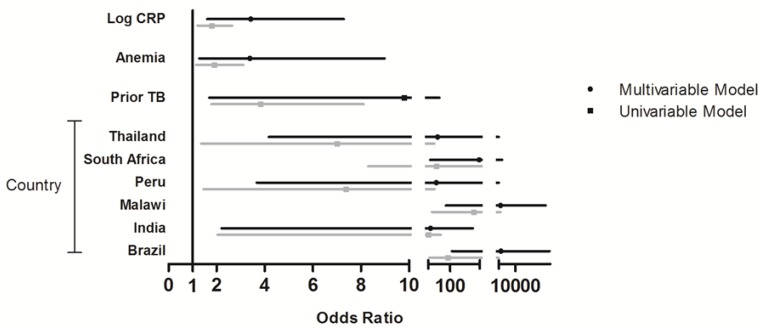
Independent factors associated with serum selenium deficiency. Forest plot showing the odds ratio (OR) of being selenium deficient (based on serum cutoffs) by covariates. Univariable and multivariable logistic regression were used to calculate the OR of being selenium deficient. Variables included in the model were selenium, gender, age, body mass index (BMI), country, prior Tuberculosis (TB), anemia, albumin and log C-reactive protein (CRP). Reference groups for categorical variables were male for gender, Haiti for country, no anemia and no prior TB. Zimbabwe and USA are excluded due to everyone and no one being deficient, respectively.

Median pre-ART (baseline) serum selenium concentrations in the subcohort was 82 μg/L (IQR: 57–100) and the levels varied widely by country. The prevalence of serum selenium deficiency, as defined as serum concentrations of <85 μg/L, was 53%. Gender (*p* = 0.02), country (*p* < 0.001), race (*p* = 0.007), prior tuberculosis (TB) (*p* < 0.001), hemoglobin (*p* = 0.008), albumin (*p* < 0.0001) and log_10_ CRP (*p* = 0.006) were significant risk factors for baseline serum selenium deficiency (Table 1). Females were more likely to be deficient than males (OR: 1.86, 95% CI: 1.13–3.08). Prevalence of deficiency varied widely between countries ranging from 0% (US) to 100% (Zimbabwe) and relative to Haiti (with 7% deficiency) persons from Malawi, Brazil, and South Africa had the highest odds of being selenium deficient (Figure 2). Anemia (OR: 1.89, 95% CI: 3.11–1.14), lower concentrations of albumin (OR: 4.46, 95% CI: 2.52–7.86), and higher levels of log_10_ CRP (OR: 1.79, 95% CI: 1.20–2.65) were also associated with greater serum selenium deficiency.

**Table 1:** Characteristics of study population by serum selenium deficiency (<85 μg/L) status.

Characteristic		Selenium Levels			*p*-Value
	All (*n* = 252) *n* (%)	All (*n* = 252) Median (IQR)	Deficient (*n* = 134) *n* (%)	Normal (*n* = 118) *n* (%)	
**Gender**					
Male	131 (52)	88 (58–107)	60 (46)	71 (54)	0.02
Female	121 (48)	78 (57–95)	74 (61)	47 (39)	
Age, median (IQR)	35 (30–41)		35 (30–41)	34.5 (29–41)	0.26
**Country**					
Brazil	30 (12)	53 (46–61)	26 (87)	4 (13)	<0.001
Haiti	30 (12)	109 (99–126)	2 (7)	28 (93)	
India	18 (7)	88 (67–106)	8 (44)	10 (56)	
Malawi	30 (12)	58 (49–73)	28 (93)	2 (7)	
Peru	29 (12)	92 (83–101)	10 (34)	19 (66)	
South Africa	29 (12)	73 (67–84)	22 (76)	7 (24)	
Thailand	27 (11)	93 (84–97)	9 (33)	18 (67)	
US	30 (12)	118 (108–128)	0 (0)	30 (100)	
Zimbabwe	29 (12)	50 (41–59)	29 (100)	0 (0)	
**Race**					
White	15 (6)	103 (46–125)	7 (47)	8 (53)	0.007
Black	133 (53)	73 (54–99)	84 (63)	49 (37)	
Hispanic	57 (23)	87 (67–101)	26 (46)	31 (54)	
Asian	46 (18)	91 (79–97)	17 (37)	29 (63)	
**Body mass index (kg/m^2^)**					
<18.5	19 (8)	63 (45–97)	13 (68)	6 (32)	0.16
18.5–25	166 (66)	83 (57–99)	91 (55)	75 (45)	
≥25	67 (27)	88 (67–108)	30 (45)	37 (55)	
**Treatment**					
A	94 (37)	84 (57–96)	48 (51)	46 (49)	0.61
B	83 (33)	76 (58–100)	48 (58)	35 (42)	
C	75 (30)	85 (59–104)	38 (51)	37 (49)	
Prior TB diagnosis	45 (18)	84 (57–96)	45 (18)	35 (78)	<0.001
CD4 count, median (IQR), cells/μL	179 (88, 231)		179 (88, 231)	171 (71, 233)	0.53
Viral load, median (IQR), log_10_ copies/mL	5.1 (4.6, 5.5)		5.1 (4.7, 5.5)	5.0 (4.5, 5.5)	0.26
Hemoglobin, median (IQR), g/dL	12.4 (10.9, 13.8)		12.1 (10.6, 13.7)	12.8 (11.4, 14.1)	0.008
Albumin, median (IQR), g/dL	3.9 (3.6, 4.3)		3.8 (3.3, 4.1)	4.1 (3.9, 4.4)	<0.0001
Log CRP, median (IQR), mg/L	3.4 (1.4, 10.3)		5.0 (1.4, 16.2)	2.4 (1.2, 7.1)	0.006

Data are presented from the random subcohort (*n* = 252 with selenium data). The first column gives the total number (%) of people in the subcohort within each category of a covariate; the second column gives median serum selenium concentration by covariates; in the third and fourth column, data are represented as number (%) of people in the subcohort that are either selenium deficient or normal within each category of a covariate. For continuous covariates, the median and interquartile range (IQR) values of the covariate is presented for selenium deficient and normal; Wilcoxon’s rank sum test for continuous variables and Fisher’s exact test for categorical variables were used to calculate the *p*-values shown in the last column to compare those with and without serum selenium deficiency in the subcohort. Units for serum selenium concentration is μg/L and serum selenium deficiency is defined as <85 μg/L. Abbreviations: IQR: Interquartile range; OR: Odds Ratio; CI: confidence intervals; CRP: C-reactive protein. Treatments A: efavirenz plus twice-daily lamivudine-zidovudine; B: atazanavir plus didanosine EC and emtricitabine all given once daily; C: efarivenz plus emtricitabine-tenoforvir-DF once daily.

After adjusting for sex, age, BMI and albumin, independent risk factors for selenium deficiency were country, previous TB (adjusted odds ratio (aOR): 9.79, 95% CI: 1.69–56.83), anemia (aOR: 3.38, 95% CI: 1.27–8.99) and log_10_ CRP (aOR: 3.42, 95% CI: 1.60–7.29) in multivariable logistic regression models (Figure 2). Being from Malawi, Brazil and South Africa also had the highest odds of being deficient relative to Haiti in multivariable models (Figure 2). Adjusted OR could not be calculated for Zimbabwe as everyone was deficient and for the US as no one was deficient.

### 3.3. Association of Baseline Serum Selenium with Clinical Failure and Virologic Failure

There were 193 cases (46.7%) in the full case-cohort that had clinical treatment failure defined as WHO stage 3 (*n* = 129 including 45 pulmonary TB), 4 (*n* = 49 including 13 extrapulmonary TB) or death (*n* = 15). Serum selenium deficiency (<85 μg/L) was not associated with increased hazards (hazard ratio (HR): 1.85, 95% CI: 0.92–3.7) of clinical treatment failure in univariable analyses (Table 2a). In multivariable model adjusting for gender, age, BMI, CD4 count, viral load, hemoglobin and albumin (Multivariable model 1), serum selenium deficiency was not associated (aHR: 1.43, 95% CI: 0.68–3.02) with clinical failure either (Table 2a). Further adjusting for inflammation (CRP) (Multivariable model 2) did not change the lack of association (aHR: 1.14, 95% CI: 0.49–2.61) between serum selenium deficiency and clinical failure (Table 2a).

**Table 2:** (**a**) Association of baseline serum selenium concentrations with clinical treatment failure (*n* = 413); (**b**) Association of baseline serum selenium concentrations with virologic failure (*n* = 381). Univariable and multivariable cox proportional hazards regression models were used to calculate the hazard ratios to assess the association of serum selenium with clinical treatment failure (**a**) and virologic failure (**b**) in the full case-cohort. The outcome of interest was clinical failure (primary outcome) or virologic failure (secondary outcome). For the univariable and multivariable analyses of clinical failure and virologic failure, serum selenium was categorized as either a binary variable (deficient or not) or as quartiles with the third quartile as the reference.

a				
	*n* (%)	Univariable	Multivariable 1 (Adjusted for Gender, Age, BMI, CD4 Count, Viral Load, Hemoglobin and Albumin)	Multivariable 2 (Adjusted for Variables in Multivariable Model 1 + CRP)
**By selenium deficiency status**				
Normal (≥85 μg/L)	173 (42)	Reference	Reference	Reference
Selenium Deficient (	240 (58)	1.85 (0.92–3.70)	1.43 (0.68–3.02)	1.14 (0.49–2.61)
**By quartiles of selenium**				
Quartile 1 (	98 (24)	3.51 (1.06–11.60)	2.71 (0.70–10.45)	2.25 (0.57–8.79)
Quartile 2 (55.60–75.52 μg/L)	94 (23)	2.57 (1.04–6.35)	1.74 (0.60–5.03)	1.46 (0.49–4.35)
Quartile 3 (75.52–97.45 μg/L)	111 (27)	Reference	Reference	Reference
Quartile 4 (>97.45 μg/L)	110 (26)	2.16 (0.88–5.30)	3.50 (1.30–9.42)	4.13 (1.65–10.35)
**b**				
	***n* (%)**	**Univariable**	**Multivariable 3 (Adjusted for Gender, Age, BMI, CD4 Count, Viral Load, Treatment Arm)**	**Multivariable 4 (Adjusted for Variables in Multivariable Model 3 + CRP)**
**By selenium deficiency status**				
Normal (≥85 μg/L)	221 (58)	Reference	Reference	Reference
Selenium Deficient (<85 µg/L)	160 (42)	0.88 (0.43–1.8)	0.68 (0.31–1.48)	0.64 (0.28–1.46)
**By quartiles of selenium**				
Quartile 1 (<55.73 µg/L)	89 (23)	1.37 (0.47–3.98)	1.32 (0.45–3.91)	1.29 (0.44–3.79)
Quartile 2 (55.73–74.86 μg/L)	85 (23)	1.11 (0.50–2.51)	0.97 (0.43–2.22)	0.91 (0.39–2.13)
Quartile 3 (74.86–95.10 μg/L)	99 (26)	Reference	Reference	Reference
Quartile 4 (>95.10 μg/L)	108 (28)	0.84 (0.31–2.33)	0.92 (0.34–2.50)	0.87 (0.30–2.49)

Interestingly, we observed that when serum selenium quartiles were used as the exposure variable (with quartile 3 as the reference) in univariable models, the lowest quartile (HR: 3.51, 95% CI: 1.06–11.60) and quartile 2 (HR: 2.57, 95% CI: 1.04–6.35) were associated with clinical treatment failure while there was a trend for association with the highest quartile (HR: 2.16, 95% CI: 0.88–5.30) (Table 2a). In multivariable model 1, there was also an increased hazards for the lowest quartile (aHR: 2.71, 95% CI: 0.70–10.45) and quartile 2 (aHR: 1.74, 95% CI: 0.60–5.03) relative to quartile 3, but this association was not statistically significant (Table 2a). However, the highest quartile had a statistically significant hazards (aHR: 3.50, 95% CI: 1.30–9.42) (Table 2a) of clinical failure. This association of the highest quartile (relative to third quartile) of serum selenium with clinical failure remained even after adjusting for CRP (multivariable model 2 in Table 2a) as well as after adjusting for other antioxidants (retinol and Vitamin E) (data not shown). Given that clinical features of WHO stage III and death (both defined as clinical failure here along with WHO stage IV) are quite different, we tested and confirmed the association of highest quartile of selenium with outcome (aHR: 3.47, 95% CI: 1.41–8.52) even when we limited our clinical failure definition to WHO stage III and IV (excluding death). People from Haiti, India and USA were more likely to be in the highest quartile of serum selenium (data not shown) and accounted for most (98%) of the cases in the highest quartile.

To determine the association of baseline serum selenium concentrations and virologic failure, we also categorized selenium as either a binary variable or as quartiles. Neither serum selenium deficiency nor any of the serum selenium quartiles were associated with virologic failure in univariable or multivariable models (Table 2b).

## 4. Discussion

In our multicenter study, prevalence of serum selenium deficiency varied significantly among HIV-infected people residing in diverse regions of the world. Baseline serum selenium deficiency pre-ART was associated with country of origin, anemia, CRP concentrations, and previous TB. Pre-ART serum selenium deficiency was not associated with clinical or virologic treatment failure. Interestingly, we observed that serum selenium concentrations in the highest quartile were predictive of increased clinical failure compared to people in the third quartile. While other studies have shown that selenium deficiency is associated with natural HIV disease progression and severity in the pre-ART era, our study is the first to show that high baseline serum selenium concentrations among HIV-infected people initiating ART is associated with the adverse treatment outcome of clinical failure. Our findings have important implications for programs considering selenium supplementation strategies among HIV-infected adults initiating ART.

We found that the prevalence of serum selenium deficiency varied greatly between countries, ranging from 0% to 100%. Unlike many other micronutrients, selenium levels in humans do not necessarily correlate with malnutrition [11] (and as seen with the lack of association with BMI in our study) but rather on the differences in the content of selenium in the soil, the varying bioavailability of selenium in diverse foods, use of micronutrient supplements and the access to imported food from other regions of the world [17,27].

Other risk factors for baseline serum selenium deficiency were anemia, higher CRP concentrations and prior TB. Low serum selenium has previously been associated with anemia and potential biological mechanisms include low selenium affecting inflammation to cause anemia of inflammation or low selenium increasing oxidative stress that leads to erythrocyte damage [28]. The inverse association of CRP with serum selenium levels in our study has also been observed by other groups in both non-HIV [26,29,30] and HIV settings [19,31]. However, as selenium and inflammation have a bi-directional relationship [11,18], it is possible that CRP might not be a risk factor but a result of selenium deficiency as deficiency can also lead to changes in inflammation. Individuals with replete selenium could potentially control inflammation and the acute phase response (hence CRP) by inhibiting NF-κβ signaling [26].

Previous cross-sectional and longitudinal HIV natural history studies in the pre-ART era have shown an association between low serum selenium concentrations and progression of infection, morbidity and mortality [19,32,33,34,35,36,37]. Low serum selenium concentration has also been associated with HIV progression among persons already on ART but studies assessing baseline selenium status and post-ART initiation outcomes have been lacking [23,24,38,39,40,41,42,43,44,45,46]. In two randomized controlled trials (RCT) of HIV-positive people in the US, selenium supplementation reduced viral load and hospitalizations but in these studies no one had selenium deficiency and the majority of people were already on ART [8,9]. In a third RCT of selenium supplementation of HIV-positive pregnant women (only ~2% were selenium deficient) in Tanzania where minimal beneficial effects of supplementation on CD4 count, viral load and mortality were seen, the vast majority of participants (4%) did not receive ART during the study [7]. To our knowledge, this is the first study to longitudinally follow HIV-positive patients initiating ART to study the association of baseline pre-ART serum selenium concentrations with progression to clinical treatment failure post-ART.

We did not observe serum selenium deficiency to be associated with clinical treatment failure. However, we observed an increased clinical failure in people with the highest quartile of serum selenium concentrations. While this was initially surprising as our hypothesis was that more failure would be observed in people with selenium deficiency, the result we found is biologically plausible. A U-shaped relationship has been observed where both high and low selenium status have been associated with all-cause mortality and cancer in US populations [11]. This is consistent with the idea that a narrow range of therapeutic benefit exists for many micronutrients [47] and that too much selenium might result in adverse outcomes. Importantly, this association was observed even after controlling for CRP because it has been suggested that not adjusting for acute phase response (example: CRP) has been one of the weaknesses of studies looking at selenium and HIV disease outcomes [11,19]. Whether inflammation is a confounder or a result of selenium deficiency, the association of high serum selenium with clinical failure remains the same.

The potential adverse role of elevated selenium could be explained by looking at the role of selenium as a component of many antioxidants. While pro-oxidants are often thought of as harmful and antioxidants as beneficial, it is crucial to understand that both of them serve important biological functions and a balance between pro-oxidants and antioxidants is necessary [48]. When there is an imbalance with an excess of pro-oxidants, damage can be done to crucial cellular macromolecules including DNA, RNA and proteins [49]. However, a lack of pro-oxidants and an excess of antioxidants could adversely affect necessary functions such as cell death, inflammation and aging [48]. As oxidative stress is increased during both HIV infection as well as ART [14], it is possible that for people with high selenium, there is a dysregulation of oxidative stress resulting in worse treatment outcomes. Importantly, this association of elevated serum selenium with clinical failure did not change even after controlling for vitamin A and vitamin E (other antioxidants).

Based on our quartile values, the group with the highest hazards of clinical failure were people with baseline serum selenium concentrations of greater than 97 μg/L while the group with the lowest hazards were people with concentrations between 75 and 97 μg/L. A potential reason that high serum selenium, but not serum selenium deficiency (<85 μg/L), was associated with greater failure might be because there are different ranges for selenium needed for optimal expression of different selenoproteins [50,51]. Thus, it could be possible that too much activity (seen at high levels) of a certain selenoprotein important during HIV and ART might result in dysregulation of oxidative stress and worse treatment outcomes.

Lastly, we did not find any association between selenium deficiency and virologic failure similar to a recent study conducted in Thailand but in contrast to a selenium supplementation trial conducted in the US, which had adults predominantly on ART and without evidence of selenium deficiency [8,52]. The results of the US RCT study, however, were controversial [53,54,55] due to potential issues with study design and interpretation of results. A new trial by the same group in Botswana [10] showed that the viral load was not different (although CD4 levels were) in ART-naïve HIV-infected people after concurrent supplementation with multivitamins (defined as Vitamin B, C and E) and selenium as compared to placebo, multivitamins alone or selenium alone. The discordant findings between the association of selenium with clinical failure as compared to virologic failure could potentially be that selenium causes clinical failure through other mechanisms such as chronic inflammation or dysregulation of oxidation reactions without affecting the viral load.

Our study did have some limitations. While we were able to estimate country specific selenium deficiency prevalence and observed some substantial differences by country, we were not sufficiently powered to look at the independent effects of individual country and selenium interactions on clinical or virological outcomes. However, this study still gives us valuable biological information on serum selenium concentrations (irrespective of country) and response to treatment. Another limitation of our study is that serum selenium concentrations, while widely used to define deficiency in previous HIV studies, might not reflect true selenium status of the whole body as well as at a cellular level (as glutathione peroxidase would have). We also did not account for social factors in our study including substance abuse and criminalization that can affect treatment failure, however these factors usually affect treatment adherence and are likely not as important to our study as the patients had relatively high treatment adherence [56]. Finally, the association we observe cannot determine causality. However, we provide valuable information for future studies and randomized trials on the potentially narrow therapeutic range of selenium and probable need for different recommendations by country or selenium status in the population.

## 5. Conclusions

In conclusion, high concentrations of serum selenium pre-ART was associated with increased clinical failure in our multi-country study. Supplementation of selenium for HIV-infected people initiating ART should be handled with caution, as we need further studies in diverse settings to determine the range of selenium in the population and the effect of low or high selenium on ART outcomes.
